# Transcriptional and Epigenetic Regulation of Effector and Memory CD8 T Cell Differentiation

**DOI:** 10.3389/fimmu.2018.02826

**Published:** 2018-12-07

**Authors:** Yao Chen, Ryan Zander, Achia Khatun, David M. Schauder, Weiguo Cui

**Affiliations:** ^1^Department of Microbiology and Immunology, Medical College of Wisconsin, Milwaukee, WI, United States; ^2^Blood Center of Wisconsin, Blood Research Institute, Milwaukee, WI, United States

**Keywords:** CD8 T cell, memory differentiation, cell fate decision, transcriptional, epigenetic, single cell sequencing

## Abstract

Immune protection and lasting memory are accomplished through the generation of phenotypically and functionally distinct CD8 T cell subsets. Understanding how these effector and memory T cells are formed is the first step in eventually manipulating the immune system for therapeutic benefit. In this review, we will summarize the current understanding of CD8 T cell differentiation upon acute infection, with a focus on the transcriptional and epigenetic regulation of cell fate decision and memory formation. Moreover, we will highlight the importance of high throughput sequencing approaches and single cell technologies in providing insight into genome-wide investigations and the heterogeneity of individual CD8 T cells.

## Introduction

During an acute viral or bacterial infection, pathogen-specific T cells robustly proliferate, acquire effector functions, and migrate to the site of infection to eliminate the pathogen. The majority (>90%) of antigen-specific CD8 T cells die via apoptosis upon pathogen clearance, leaving behind distinct memory subsets with unique phenotypic and functional properties. However, the molecular and genetic mechanisms that guide how these cell fate decisions are made remains incompletely understood. Additionally, although it is well-appreciated that antigen specific memory CD8 T cells can persist for extended periods of time in a functionally quiescent state, and that this is important for conferring long-term protective immunity against previously encountered pathogens, the underlying mechanisms that endow memory CD8 T cells with this longevity remain unclear. Moreover, the molecular pathways that help maintain the phenotypic and functional heterogeneity of memory subsets, and enable memory CD8 T cells to remain poised to quickly recall their effector function are still incompletely understood. Current evidence suggests that multiple signals, such as T-cell receptor (TCR), co-stimulation, inflammation, and metabolic signals can orchestrate CD8 T cell fate decisions, with some of these commitment choices occurring early in the immune response (1, 2). As the incorporation of multiple distinct signals received by individual T cells likely triggers diverse transcriptional programs, it is important to discuss the key transcription factors that have been known to orchestrate CD8 T cell fate decision. Moreover, we highlight the field's current understanding of CD8 T cell differentiation on the epigenetic and single-cell level, and provide a brief discussion on how modern technologies may help to refine the CD8 T cell differentiation paradigm.

## Memory CD8 T Cell Differentiation and Cell Fate decision

The process of memory CD8 T cell selection is not entirely stochastic, as originally proposed (3), as effector cells can display inherently distinct memory cell potential, with some CD8 T cells being intrinsically better at persisting and populating the memory pool. It was previously identified that a small subset of effector T cells survive the contraction phase and serve as the precursors of the memory CD8 T cell compartment (4–8). This minor population of effector cells, termed memory precursor effector cells (MPECs), can be distinguished based on their high expression levels of CD127, the IL-7 receptor alpha (IL-7Rα), and their decreased expression of killer cell lectin-like receptor G1 (KLRG1) (5, 6). Other surface proteins that co-segregate with increased IL-7Rα expression on MPECs include CD27, CD28, CD62L, and CXCR3 (1). By contrast, a larger proportion of effector CD8 T cells display high expression of KLRG1 and low expression of IL-7Rα and are more terminally differentiated than their MPEC counterparts. This subset of KLRG1^hi^ effector CD8 T cells is collectively referred to as short-lived effector cells (SLECs). Of note, although MPECs and SLECs were observed in various infectious settings in different species including humans, these phenotypic distinctions are not exclusive criteria for forming memory T cells nor do they represent universal markers for memory precursor cells across all types of immune response (9–11). Furthermore, several studies previously demonstrated that MPECs can give rise to both T central memory (T_CM_) and T effector memory (T_EM_) populations (1, 5–7) and recent evidence further indicates that the precursors of tissue resident memory cells (T_RM_) in the skin and small intestine are also derived from less differentiated KLRG1^lo^ memory T cell precursor cells (12, 13). It is important to note however that these phenotypic distinctions are not exclusive criteria for memory T cell formation, as cell death may also occur among IL-7Rα^hi^ effector T cells following infection, and many long-lived KLRG1^hi^IL-7Rα^hi^ memory CD8 T cells have been observed following secondary infections (14–17). Moreover, the frequency of KLRG1^hi^ cells can vary widely depending on the type of infection or vaccination. Indeed, a recent study further highlighted the limitations of these markers and elegantly demonstrated that some KLRG1^hi^ cells can downregulate KLRG1 during the contraction phase and differentiate into all memory T cell lineages (18). Thus, a higher degree of developmental plasticity than previously appreciated may exist during the effector to memory CD8 T cell transition phase. Importantly, however, these cell surface markers do offer a useful framework of determining the relative memory cell potential of effector CD8 T cells in several circumstances, and they have become invaluable for identifying molecular pathways that regulate these effector-to-memory cell fate decisions.

### Heterogeneity of Memory CD8 T Cell Subsets

As CD8 T cells transition from naïve to effector to memory cells, their overall gene expression profiles changed, resulting in phenotypic and functional variations among the different populations. As such, several fundamental studies have demonstrated that memory CD8 T cells can be compartmentalized into at least 3 distinct subsets on the basis of their effector function, proliferative potential, migration patterns and transcriptional program (19–23). For well over a decade, the population of circulating memory CD8 T cells has been broadly categorized into two distinct subsets, conventionally designated T_CM_ and T_EM_ (20, 24). These two subsets can be distinguished based on their differential expression of CCR7 and CD62L (L-selectin), with T_CM_ cells expressing both of these lymph node homing receptor molecules which facilitates their trafficking to and retention within secondary lymphoid tissues (19, 20). By contrast, T_EM_ cells lack expression of CCR7 and CD62L and are most commonly found in the blood and in non-lymphoid tissues (e.g., lung, liver, intestine) (20, 21, 25). Compared to T_EM_, T_CM_ cells display an enhanced proliferative potential and an increased capacity to produce the cytokine IL-2, but are unable to immediately produce effector molecules until they undergo secondary proliferation and differentiate into effector cells (20, 26–28). Conversely, T_EM_ cells constitutively display effector functions such as cytolytic activity and IFN-γ production (1, 21, 29, 30). Notably, within the past 10 years, T_RM_ have emerged as the third major memory CD8 T cell subset and have been identified to permanently reside in peripheral tissues after pathogen clearance and provide site-specific protection upon re-infection (22, 23). The specific anatomical location of where T_RM_ cells develop and are maintained can depend on the nature or route of the infection and the inflammatory signals experienced during the effector phase of the T cell response (31). T_RM_ cells can generally be distinguished from T_EM_ cells infiltrating non-lymphoid tissues based on their high expression of CD69 and the integrin CD103 (12, 32–35), although not all T_RM_ cells constitutively express CD103 (31, 34). An important component of T_RM_ differentiation is the migration of T effector cells to target sites (such as the skin or intestines) and their subsequent downregulation of tissue egress receptors, such as S1PR1 (35) and upregulation of adhesion molecules, such as CD103 (12). Other distinguishable features of T_RM_ cells are their sustained expression of granzyme B (that may vary by location) and their maintained high levels of mRNAs encoding TNFα, IFNγ, and IL-2 (31), which allow them to eliminate any re-occurring microbial threat at portal entry sites. Whether T_RM_ undergo homeostatic proliferation to maintain a stable population has not been clearly demonstrated. T_RM_ cells from brain, skin and mucosal sites showed much lower homeostatic proliferation ability and turnover rate compare to their circulating counterparts (22, 31, 36, 37). Interestingly, T_RM_ cells in the lung airway may require constant replenishment from recirculating memory cells (38). As T_RM_ and T_EM_ subsets display constitutive effector functions and occupy the frontline sites of pathogen entry, they are uniquely positioned to be among the first responders of the adaptive recall response. Conversely, T_CM_ recall is critical for the rapid generation of a pool of secondary effector cells that may help contain pathogens that breach the initial containment. In humans and mice, there is a newly defined subset, called T memory stem (T_SCM_) cells. The characteristic of T_SCM_ is stemness. T_SCM_ cells represent increased proliferative, self-renewal and long-term persistence capacity (39, 40). Additionally, only naive T cells and T_SCM_ cells were able to reconstitute the entire heterogeneity of memory T cell subsets, indicating that T_SCM_ cells are multipotent (39). In patients undergoing haploidentical hematopoietic stem cell transplantation (HSCT), T_SCM_ cells are preferentially generated from naïve cells and the dominant long-term clonotypes appeared to preferentially originate from infused T_SCM_ rather than T_CM_ clones (41, 42). Gene expression data also showed that there is a progressive change moving from naïve to T_SCM_ to T_CM_ and T_EM_ cells (39). These evidences indicate that T_SCM_ cells are at the apex of the hierarchical tree of T cell differentiation and at a hierarchically superior level over the T_CM_ cells (40). Moreover, memory T cells can be further subdivided based on differential expression of additional phenotypic markers. As one example of such endeavor, CX_3_CR1 has been recently used to identify a peripheral memory (T_PM_) subset that possesses high cytotoxicity and provides global immune surveillance (43, 44). Collectively, the formation of these distinct memory CD8 T cell subsets and their division of labor likely ensures optimal protective immunity upon pathogen re-challenge. However, a key question that remains to be addressed is whether these distinct memory subsets are maintained by signals from the tissue microenvironment or preprogrammed by cell-intrinsic mechanisms, such as transcription profiles and the chromatin landscape.

### The Impact of Signal Strength on CD8 T Cell Fate

**Figure 1:**
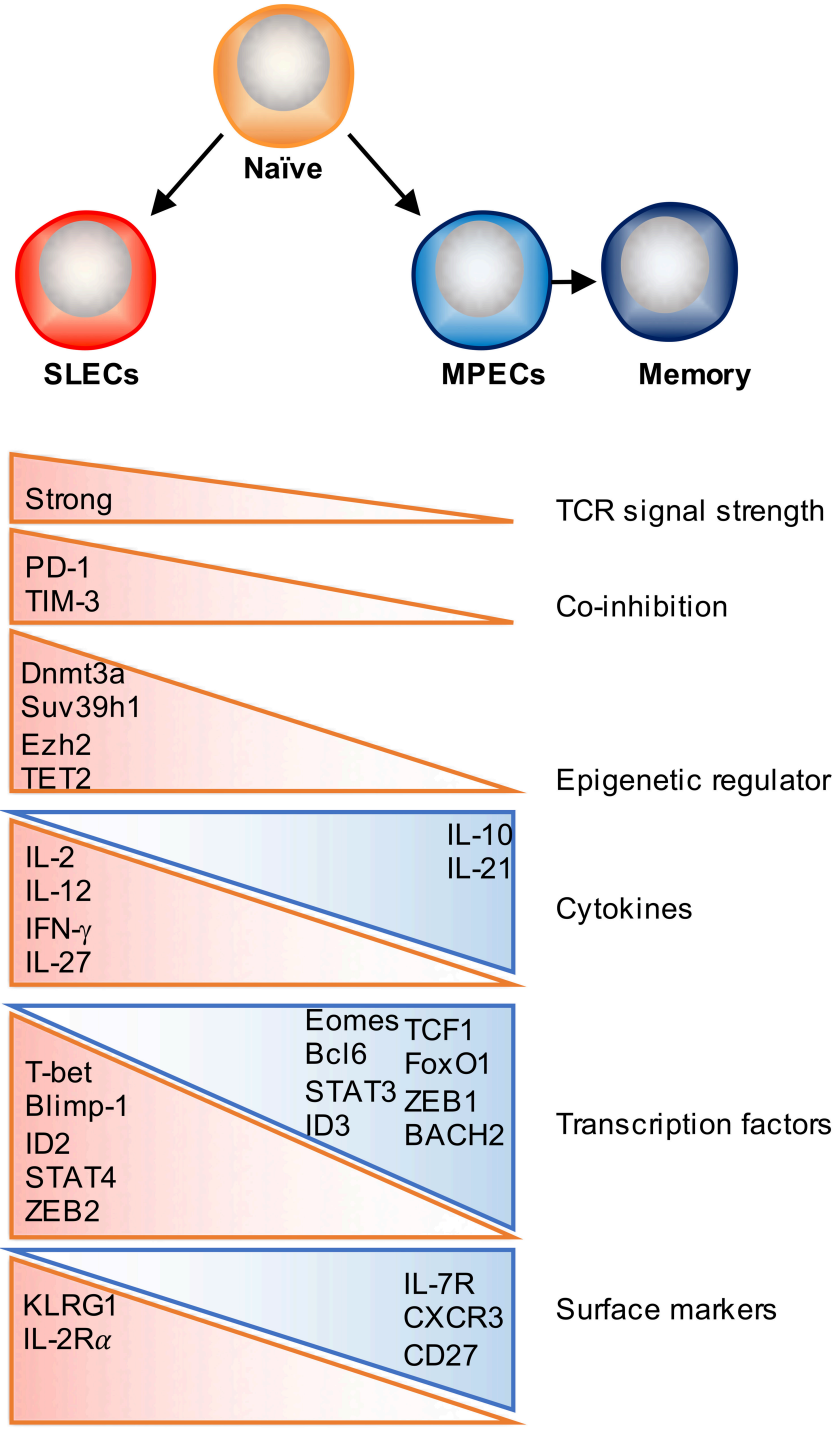
Factors that regulate effector and memory cell fate decision. Following activation, antigen-specific naïve CD8 T cells proliferate and differentiate into a heterogeneous pool of effector T cells that consist of two major subsets: SLECs and MPECs. Majority of SLECs die by apoptosis during contraction phase, whereas MPECs survive and become long-lived memory cells. Numerous factors as depicted can contribute to this cell fate decision process, which include TCR signal strength, co-stimulatory/co-inhibitory molecules, cytokines, transcription factors, and epigenetic regulators.

During infection or vaccination, naïve CD8 T cells engage with antigen-presenting dendritic cells (DCs) and are presented cognate peptide in a major histocompatibility complex (MHC) class 1-restricted manner (45, 46). Upon TCR-mediated recognition of the MHC-peptide complex, antigen-specific CD8 T cells will start to rapidly proliferate and acquire effector functions and the ability to migrate to sites of infection. During this process of T cell priming, newly activated T cells will integrate multiple signals in the form of TCR signaling, co-stimulation, cytokine, chemokine, and metabolic signals, all of which can have a major impact on the accumulation, survival, and cell-fate decision of effector T cells (1, 2, 47) (Figure 1).

TCR signaling is one of the initiating signals that helps shape T cell memory. The strength and quality of TCR signaling, which is determined by the affinity of the TCR for peptide–MHC molecules (pMHC), the dose of antigen presented by APCs, the duration of the TCR–pMHC interaction, and the timing of TCR recognition (early or late during infection phase) have been shown to partially contribute to memory commitment, function and the diversity of the memory pool (48). The balance between co-stimulatory and co-inhibitory signaling is not only required for effector T cell activation and expansion, but also determines the size and quality of the memory T cell pool (49). Co-stimulatory molecules, such as CD28, 4-1BB, CD27 and OX40 have been shown to promote memory formation as well as contribute to secondary responses (50, 51). As for co-inhibitory signals, it has previously been reported that lower PD-1 expression may drive T cell differentiation away from a SLEC fate and skew toward T_EM_ memory generation (52). TIM-3 is another inhibitory receptor and blockade of TIM-3 increases transcription of genes involved in T cell effector function and differentiation but decreases expression of genes associated with memory T cell formation (52, 53). Further studies are required to determine how co-stimulatory and co-inhibitory signaling pathways coordinately regulate memory T cell development.

Several studies have identified that exposure to certain inflammatory signals can play a major role in regulating the differentiation of effector and memory CD8 T cell subsets in a context dependent manner. For example, IL-12 or IL-27 enhances SLEC formation during acute bacterial or viral infection, whereas type I and type II interferons can either promote memory or enhance SLEC differentiation under different settings (17, 54–59). Other studies have further identified that exposure to IL-15 helps skew CD8 T cells along a memory pathway (60–62), whereas IL-2 signaling is implicated in promoting the differentiation of short-lived effector T cells (63, 64). However, the effects of IL-2 signaling on CD8 T cell memory formation may be regulated in a temporal manner, as administration of recombinant IL-2 (rIL-2) during the expansion phase diminishes T cell survival, whereas treatment with rIL-2 during the contraction phase promotes T cell proliferation, survival, and memory formation (65). By contrast, IL-10 and IL-21 signaling through a STAT3-SOCS3 pathway was found to promote memory formation, potentially by insulating T cells from excessive inflammatory stimuli (66). Recent studies have also begun to shed light on potential cytokine signaling pathways that contribute to T_RM_ development, with recent findings elucidating an important role for the cytokine transforming growth factor β (TGF-β) and IL-15 in facilitating T_RM_ differentiation by inducing CD103 expression on T_RM_ precursor cells infiltrating the skin, lung, and small intestine (12, 13, 34, 67, 68).

## Transcriptional Regulation of Effector and Memory CD8 T Cell Differentiation

### Pioneer Transcription Factors Initiate Effector Differentiation

**Figure 2:**
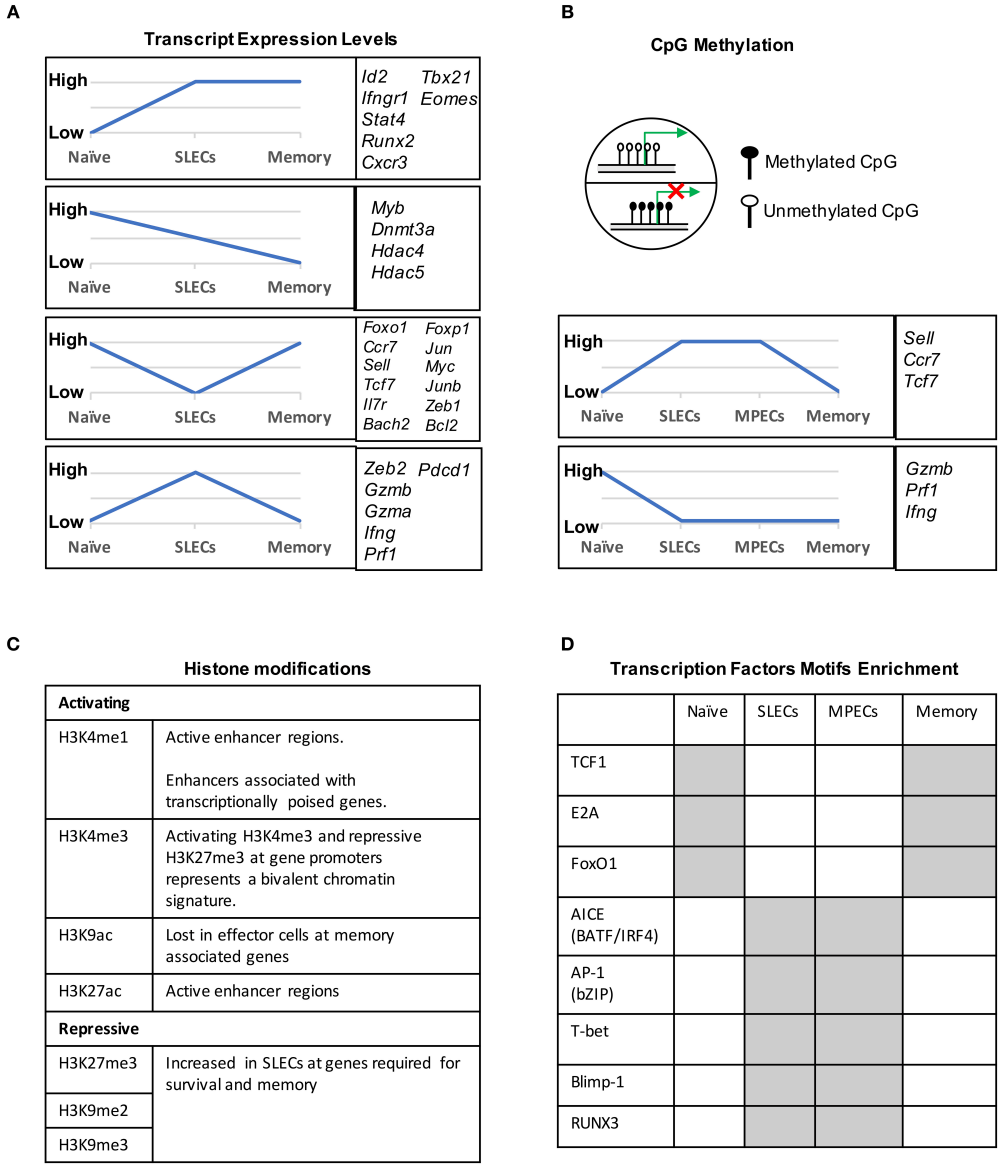
Transcriptional and epigenetic profiling during naïve to effector to memory transition. **(A)** Gene clusters defined by the mRNA expression levels in naïve, SLECs and memory cells. Four major expression patterns emerged: genes that were up- or downregulated during the effector stage and persisted into the memory phase, and genes that were up- or downregulated during the effector phase and then reverted to the naive state. **(B)** CpG methylation levels of different genes in naïve, SLECs, MPECs and memory cells. Naïve/memory genes are similarly acquired CpG methylation in both SLECs and MPECs. MPECs, not SLECs, have the capacity to erase their newly acquired methylation programs and re-express naïve/memory genes as they develop into memory CD8 T cells. SLECs and MPECs both show demethylation of several effector-associated genes which remain demethylated in memory cells for a long period of time. **(C)** Histones posttranslational modification (PTM) and their functions that are essential for CD8 T cell differentiation. For example, the epigenetic bivalency for H3K27me3 and H3K4me3 represent an epigenetic state from which a gene can be rapidly activated or repressed depending on the differentiation pathways. **(D)** Differentially enriched motifs of transcription factors in naïve, effector and memory cells. Motif analysis identified the cell-subsets specific transcription factors binding sites in enhancer or promoter regions. Gray depicts highly enriched motifs.

**Figure 3:**
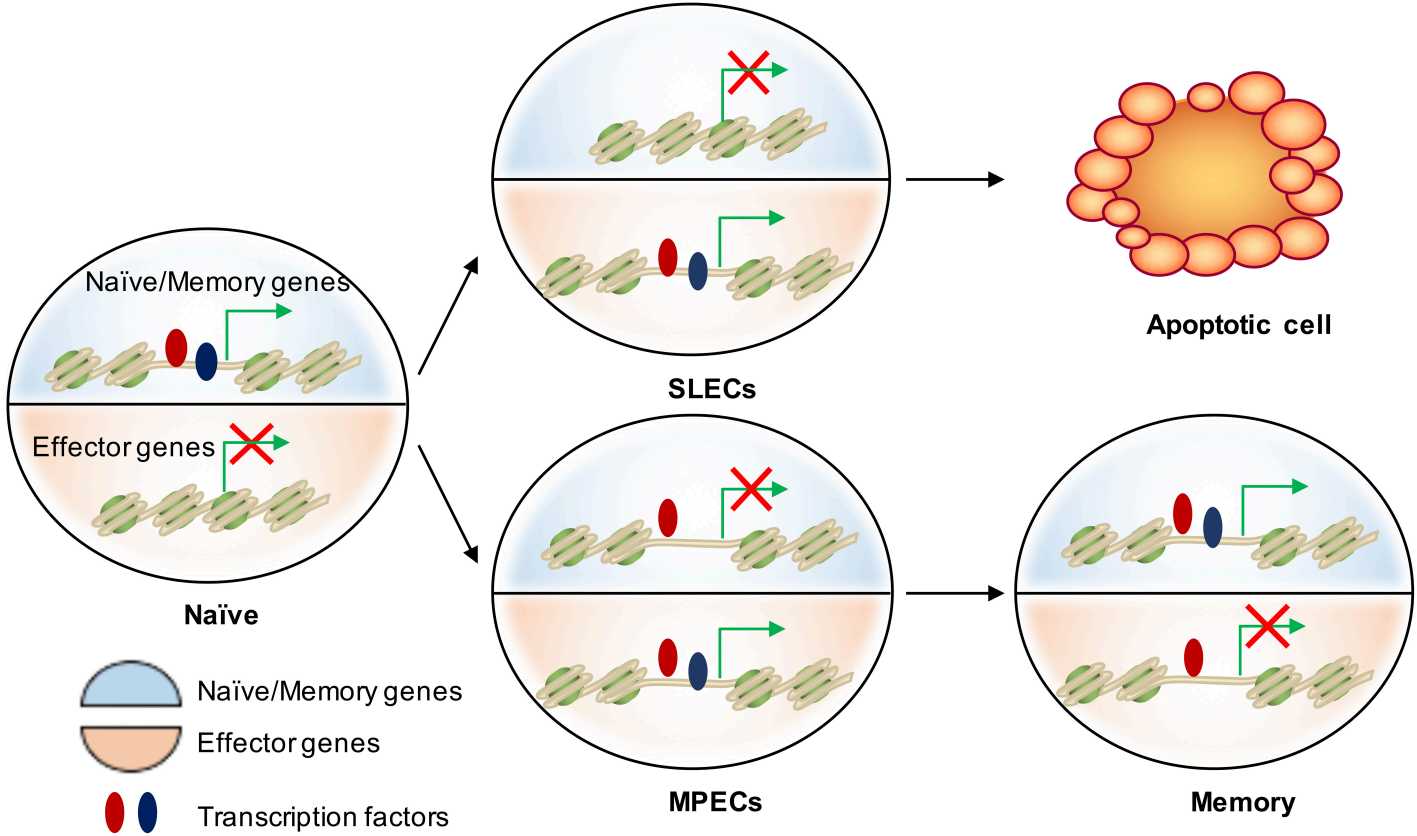
Epigenetic regulation of cell fate decision during acute infection. There are two major transcriptional circuits in regulating CD8 T cell differentiation: one of them associated with effector function and another one is essential in naïve/memory cells controlling T cell quiescence and homeostasis. In naïve CD8 T cells, naïve/memory genes are open (epigenetically by TFs in red) and on (transcriptionally by TFs in blue), while the effector genes are closed and off. When naïve cells are activated, effector genes are turned on mainly by “pioneer TFs” (in red) in both SLECs and MPECs. These genetic regions remain open but poised as MPECs develop into memory cells. Transcriptional repression of naïve/memory genes in MPECs cells can be reversed in memory CD8 T cells through recruiting additional TFs (in blue) to restart gene expression. In contrast, SLECs lose the accessibility at these TF-bound cis-regulatory elements and therefore permanently turn off the naïve/memory gene expression. This leads to their loss of memory potential and long-term survival.

During naïve to effector transition, dynamic changes occur at both the transcriptional and epigenetic level (Figures 2A, 3). Learning from CD4 differentiation (69), the fundamental identity of these heterogeneous effector CD8 T cells can generally be established by upstream “pioneer transcription factors” that regulate the entire transcriptional network to initiate early effector differentiation. Additionally, current evidence suggests that the majority gain-of-methylation and loss-of-methylation events, which represent a repressed and active transcription state respectively, happen within the first 4 days of activation, and more than half of these differentially methylated regions (DMRs) were similarly acquired in both SLECs and MPECs (70) (Figure 2B). Furthermore, effector and memory CD8 T cells have been found to share a more similar pattern of chromatin accessibility as compared to naïve CD8 T cells (71). Among these shared accessible regions, the binding motifs for bZIP, IRF, and T-box transcription factors are highly enriched (71–73) (Figure 2D). This then brings to a question, which transcription factors are initiating this early effector differentiation? Among naïve CD8 T cells, bivalency (H3K4me3^+^H3K27me3^+^) was observed at the promotors of transcription factors that are known to be crucial for initiating an effector program, such as T-bet, Eomes, Blimp1, and IRF4 (74) (Figure 2C). This finding indicates that these transcription factors may remain poised in naïve T cells but rapidly start transcription by acquiring a permissive histone methylation signature upon TCR stimulation within 24 h (74). Indeed, it has been demonstrated that IRF4 cooperates with BATF (belongs to AP-1 family) to serve as “pioneer transcription factors” that promote chromatin accessibility and gene expression associated with various aspects of effector CD8 T cell differentiation (75–78). In addition, Runx3 is another potential “pioneer transcription factor” that can initiate changes in chromatin accessibility after CD8 T cell activation, especially at the binding sites of IRF, bZIP transcription factors, and Blimp1 (73) (Figure 2D).

### Transcriptional Regulation of CD8 T Cells Fate Decisions: Terminal Differentiation or Memory Formation?

After the initial expansion phase, effector T cells can be bifurcated into two distinct effector populations, SLECs and MPECs. Early on in the effector phase, the chromatin landscape has already been universally prepared by “pioneer transcription factors,” and now lineage-specifying transcription factors start to take effect. Considering that TCR signal strength is negatively associated with memory formation (2, 48), it is possible that TCR-induced transcription factors can influence the type of progeny derived from a single T cell. One such transcription factor is IRF4, expression of which is highly dependent on the signal strength of TCR signaling (79). Indeed, IRF4 has been found to be crucial for initial expansion and promoting SLEC formation (79). In addition, the expression level of memory associated transcription factors, Eomes and TCF1 appear to be highly sensitive to graded expression levels of IRF4 both in acute and chronic viral infection, indicating potential mechanisms by which IRF4 may regulate CD8 T cell fate decision (79, 80). Furthermore, Nuclear receptor subfamily 4 group A member 1 (NR4A1) supports formation of MPECs and T_CM_ via inhibiting the expression of IRF4 by directly binding to its promoter region (81). Similarly, the transcription factor BACH2 represses genes associated with terminal differentiation by binding to their enhancer regions and attenuating the availability of AP-1 binding sites (82). In this manner, BACH2 suppresses the differentiation of SLECs and tips the balance in favor of generating memory cells (82). Collectively these findings indicate that TCR-responsive transcription factors, such as IRF4 and AP-1 family members establish effector differentiation while NR4A1 and BACH2 suppress effector-associated genes. Thus, these transcription factors may cooperatively or antagonistically regulate cell fate decisions in response to different TCR signal strength intensities.

Importantly, there is an ever-expanding list of transcription factors known to orchestrate various signals experienced during the effector phase to polarize terminal differentiation or memory formation. STATs are cytokine-induced lineage-specifying transcription factors. Inflammatory cytokines, such as IL-2, IL-12, IFN-γ, and type I IFNs, signal through STAT1, 2, 4, and 5 respectively, and direct effector CD8 T cell proliferation and differentiation by inducing T-bet and Blimp-1 expression, and downregulating Bcl6, TCF1, and IL-7Rα expression (6, 83, 84). Conversely, STAT3 activation, which is induced by IL-10 and IL-21, is necessary for memory formation by promoting the expression of memory related transcription factors, such as Bcl6, Eomes, the SOCS3 (66, 85, 86). Moreover, T-bet/Eomes, Id2/Id3 and Blimp-1/Bcl6 (1), and the newly defined ZEB1/ZEB2 axis (87, 88) have reciprocal expression patterns in SLECs and MPECs, and drive differentiation toward opposing cell fates (Figure 1). As cell identity determined by complicated gene regulatory networks, further studies should focus on how these networks cooperatively regulate downstream target genes and cell fate decisions.

### Transcription Factors Promote Memory Maintenance

Transcription factors that are critical for naïve T cell homeostasis have also been identified to promote memory CD8 T cell self-renewal and maintenance. For example, FoxO1, TCF1, and LEF1 are all highly expressed in naïve CD8 T cells, downregulated in effector cells, and re-acquired in memory cells (84, 89–93) (Figures 2A,D). Their expression is continuously required for the long-term survival and homeostatic proliferation but not the initial activation and clonal expansion, or effector function (91, 94–96). FoxO1 promotes the expression of pro-memory and pro-survival genes, such as *Il7r, Bcl2, Sell, Ccr7, Eomes, Tcf7, Bach2, Zeb1, and Socs3*, potentially by shielding these genes from deposition of repression associated histone 3 lysine 27 trimethyl (H3K27me3) chromatin modifications (91, 93). TCF1 and LEF1 are downstream factors of the Wnt-signaling pathway and their downregulation in effector cells is due to cell cycle and IL-12-dependent CpG methylation at the TCF1 promoter (84). Intriguingly, TCF1 and LEF1 can induce deacetylation at effector genes regions, such as *Prdm1*, to favor memory formation (97).

### Transcriptional Regulation of Tissue-Resident Memory CD8 T Cells

In parallel with circulating memory cell subset differentiation, T_RM_ acquire a unique transcriptional program during differentiation and adaptation to a particular microenvironment (98–101). As early as 7 days after acute infection, a unique transcriptional signature and chromatin landscape is already established in intestinal intraepithelial lymphocytes (IELs) (102). The transcription factor Runx3 has been identified as a central regulator for T_RM_ specification by controlling a core tissue-residency gene-expression program in barrier tissues (such as lung, skin, and small intestine) and non-barrier tissues (such as salivary glands and kidney), as well as in tumors (102). Blimp-1 and its homolog protein, Hobit, establish a universal transcriptional program of tissue-residency in lymphocytes, and they have been shown to be required for T_RM_ retention in the gut, skin, liver, kidneys and lung by promoting CD103 expression while repressing *Klf2, S1pr1*, and *Ccr7* expression (99). In addition, Notch controls T_RM_ maintenance by promoting CD103 expression and regulating metabolic programs (98). Recently, NR4A1 was shown to be critical in regulating the tissue residence and function of human T_RM_ (103), and AhR was also shown to be required for skin T_RM_ (104). By contrast, the transcription factors ZEB2, T-bet (87), and KLF2 (100) have been demonstrated to inhibit T_RM_ formation by promoting tissue egress. Although T-bet and Eomes can inhibit T_RM_ formation, certain levels of T-bet expression are required for CD122 expression and IL-15 mediated T_RM_ survival (105).

## The Role of Epigenetics in the Cell Fate Decision of CD8 T Cells

A critical feature of memory CD8 T cells is their ability to rapidly re-acquire effector functions upon secondary challenge with the same pathogen. We are now learning that changes in the epigenetic landscape of memory CD8 T cells, including DNA methylation, histone modifications, and chromatin accessibility, play a substantial role in this phenomenon. In this section, we will discuss how these epigenetic changes shape the effector and memory fate decision as well as memory T cell formation and function (Figure 3).

### Differences in the Epigenetic Landscapes of SLECs and MPECs Underlie Their Divergent Cell Fate Decisions

DNA methylation occurs primarily at CpG dinucleotides with the cytosine being methylated. Genomic regions with high frequencies of these CpG dinucleotide sequences are known as CpG islands and are often found in promoters. DNA methylation is commonly thought of as a repressive epigenetic mark, exerting its downstream effects by influencing transcription factor binding and acting as a docking site for various histone modifying enzymes (Figure 2B). In CD8 T cells, the DNA methyltransferase Dnmt3a has been shown to reduce MPECs formation by catalyzing DNA methylation at sites such as the promoter of *Tcf7*, a critical transcription factor for memory CD8 T cells (106). TET2 is methylcytosine dioxygenase and mediates active DNA demethylation. TET2 gene expression is rapidly and transiently induced by TCR signaling. TET2-deficient CD8 T cells rapidly acquired memory associated surface markers such as CD62L, CD27, and CXCR3 to promote memory formation (107). Interestingly, while naïve genes become methylated and effector genes become demethylated in both MPECs and SLECs, MPECs erase these DNA methylation marks at naïve genes as they develop into long-lived memory CD8 T cells, indicating that epigenetic repression in the form of DNA methylation can be reversed (70) (Figure 2B).

Genomic DNA is packaged in nucleosomes, comprised of DNA wrapped around histone octamers made up of two copies each of the histones H2A, H2B, H3, and H4. Each histone has a flexible N-terminal tail that is subject to post-translational modifications that subsequently influence transcription of nearby genes. These modifications can affect gene expression by recruiting other transcriptional regulators or, in the case of acetylation, by neutralizing the positively charged histone N-terminal tail and decreasing its interaction with negatively charged phosphates on DNA. Large-scale genomic studies have found patterns of histone modifications that can identify cis-regulatory elements such as promoters and enhancers, as well as provide information regarding their activity (108–111) (Figure 2C). Additionally, active promoters and enhancers tend to have a central region that is depleted of nucleosomes, where transcription factors can more easily access their binding sites. It is therefore reasonable to suspect that a combination of histone modifications and accessible regions also contribute to the enhanced function of memory CD8 T cells. From studies investigating chromatin accessibility using assay for transposase-accessible chromatin (ATAC)-seq (112) and the deposition of histone modifications (H3K4Me1, H3K27Ac, H3K27Me3) by chromatin immunoprecipitation (ChIP)-seq in CD8 T cells during acute infections with *Listeria monocytogenes* and lymphocytic choriomeningitis virus (LCMV), we now have a genome-wide overview of the epigenetic changes accompanying memory CD8 T cell differentiation (71, 72, 113). These studies provide important insights into the epigenetic differences between MPECs and SLECs and through which their differentiation is regulated. Regulatory regions that are more open in MPECs than SLECs are genetic loci regulate feature genes related to naïve and memory T cell properties. However, these regulatory regions are less open or permanently silenced in terminally differentiated SLECs or exhausted CD8 T cells, suggesting that MPECs keep their memory potential through maintaining accessibility at critical memory-related cis-regulatory elements (71). Terminally differentiated SLECs have increased levels of the repressive histone modification H3K27Me3 at genes required for survival and memory cell formation, and deposition of this mark is catalyzed by the polycomb repressive complex 2 (PRC2) (93). The histone methyltransferase Suv39h1 also promotes terminal differentiation by trimethylating histone H3 lysine 9 at memory-related genes, repressing their expression (114). These differences in the epigenetic landscape between the two subsets of effector CD8 T cells provides a potential mechanism for their divergent gene expression profiles and cell fate decisions.

### Epigenetic Changes in Memory CD8 T Cells Allow for Rapid Activation

The chromatin accessible regions of memory CD8 T cell are quite similar to effector cells, especially around effector gene regions (115). Moreover, their promoter regions remain demethylated from effector to memory transition (70, 115). Much work has been done investigating DNA methylation at the *Ifng* locus in CD8 T cells, which encodes the important cytokine IFNγ that is rapidly expressed by memory cells (116–120). Naïve CD8 T cells possess substantial DNA methylation at the *Ifng* promoter, at least in part due to the activity of the DNA methyltransferase Dnmt1 (117). After activation, effector CD8 T cells have this site demethylated and turn on the expression of *Ifng*. Despite no longer expressing *Ifng*, memory CD8 T cells maintain a demethylated state at the *Ifng* promoter, thereby decreasing the number of steps required before gene expression. Help from CD4 T cells during initial activation appears to play a role in this process (119). Similar patterns seem to exist at the sites of other critical CD8 T cell effector molecules, including *Gzmb* and *Prf1*, which were found to maintain their demethylated state for at least 12 years in humans who received the yellow fever virus (YFV) vaccine (115). Therefore, regulation of DNA methylation provides a mechanism for the ability of memory CD8 T cells to quickly respond to infection.

Levels of histone H3 acetylation (119, 121) and, more specifically, H3 lysine 9 acetylation (H3K9Ac) contributes to the rapid reactivation in memory CD8 T cells (122–124). Furthermore, several studies have characterized a number of different histone modifications and chromatin accessibility at a genome-wide level over the course of a CD8 T cell response to infection or vaccination (71, 74, 93, 113, 115, 125–128). In the same study mentioned earlier, YFV-specific CD8 T cells in vaccinated humans maintain open, accessible chromatin at the promoters of the effector molecules *Ifng* and *Gzmb* (115). Overall, the establishment of specific patterns of DNA methylation, histone modifications, and chromatin accessibility prime memory CD8 T cells to more rapidly produce effector molecules and clear the pathogen.

### Transcription Factors Regulating the Epigenetic Landscape of CD8 T Cells

Individual transcription factors can affect the epigenetic landscape through the recruitment of chromatin modifying enzymes or their own intrinsic activity. Blimp-1, for example, directly binds to the genes *Il2ra* and *Cd27*, recruits the histone methyltransferase G9a and the histone deacetylase HDAC2, and leads to increased deposition of the repressive marks H3K9Me2, H3K9Me3, and H3K27Me3 and decreased levels of permissive marks H3Ac and H3K4Me3 (129). The AP-1 factor BATF has been proposed to act as a pioneer transcription factor, in cooperation with its binding partner IRF4, by directly binding to tightly packed chromatin and promoting its accessibility to other transcription factors (130). Runx3 was recently shown to drive memory CD8 T cell formation by regulating chromatin accessibility of memory cell *cis*-regulatory elements (73). While TCF7 has not yet been shown to affect the epigenetic landscape during the differentiation of activated mature CD8 T cells, it establishes critical regions of open chromatin during T cell development in the thymus (131). Additionally, studies performed in thymocytes have shown that TCF7 has intrinsic histone deacetylase activity (97). Given its importance in memory CD8 T cell formation (94, 95, 132), it is likely that TCF7 uses a combination of these two methods to regulate the memory differentiation process. Other transcription factors will likely continue to be identified that either directly or indirectly lead to epigenetic changes in activated CD8 T cells, and untangling this complex network of transcription factors and the epigenetic changes they induce will help decode the differentiation of memory CD8 T cells.

## Cell Fate Determination of CD8 T Cells at Single Cell Level

Previous studies show that there can be anywhere from ~80 to 1,200 naïve CD8 T cells or from ~20 to 200 CD4 T cells specific for a particular epitope in one mouse (133, 134). Following infection, each antigen specific CD8 T cell can interpret and integrate signals in a distinct way to create differential responses in the generation of terminally differentiated effector cells and self-renewing memory T cells (6, 20). However, when and how this fate decision is made following infection has been a topic of research for many years (127, 135–139).

### Different Experimental Approaches to Study the Cell Fate of Single CD8 T Cells

In terms of fate specification from a single T cell, two obvious possibilities can happen: (1) one T cell can give rise to two daughters cells with each being capable of choosing multiple fates or (2) one T cell can give rise to daughter cells with only one fate (140). Different experimental approaches have been applied to understand the *in vivo* fate of single CD8 T cells following acute viral or bacterial infections. Using an OT-I TCR transgenic adoptive cell transfer model, it has been demonstrated that diverse cellular progeny, including both effector and memory T cells, could develop out of a single naïve T cell following infection with *L. monocytogenes* (135). Similar results have been found using tetramer enrichment to isolate antigen specific naïve CD4 T cells followed by a single cell adoptive transfer approach for the *in vivo* fate mapping for CD4 T cells (141). Surprisingly, in both cases single naïve T cells displayed diverse patterns of differentiation, yet when combined together, they resembled the endogenous T cell response in the same individual mouse. Although these studies were instrumental in developing our understanding of T cell fate decision at the single cell level, a limitation of these approaches is that they only allow for deciphering the fate of one T cell at a time per mouse. To overcome this hurdle and to facilitate the analysis of multiple T cell families at the same time, one elegant study performed adoptive transfer experiments using barcoded TCR transgenic CD8 T cells. Upon bulk transfer of single barcoded naïve CD8 T cells the authors demonstrated that individual naïve T cells have multiple fates and can differentiate into both effector and memory subsets during acute infection (136). This approach offers the opportunity to analyze large numbers of barcoded TCR transgenic single naïve CD8 T cells and their fates at the same time. However, this experimental strategy is limited by its dependency on using indirect approaches (microarray, sequencing) for barcode identification and by its inability to conduct a functional assessment of T cells at the protein level. Notably, other powerful tools have emerged that help alleviate some of these pitfalls. To serve the purpose of analyzing multiple T cell families simultaneously, adoptive transfer experiments have been accompanied with the use of a matrix co-expressing congenic markers, followed by their breeding to TCR transgenic mice (137). This innovative approach allowed for the transfer and assessment of eight naïve TCR transgenic CD8 T cells at a time and revealed differential subset diversification by each single cell resulting in broad and vigorous CD8 T cell immunity. To rule out any TCR-based influence, a limiting dilution strategy has been developed with the aim of transferring a single naïve antigen specific CD8 T cell into recipient mice, which is plausible mathematically but *in vivo* difficult to prove (138). With this approach, single naïve CD8 T cells have been found to exhibit differential cell fates as well as display some extreme bias toward a particular cell fate. Importantly, using the latest powerful technology- single-cell RNA sequencing (scRNA-seq), it has recently been demonstrated that virus-specific CD8 T cells display vast transcriptional heterogeneity and can give rise to multiple cell fates, which unexpectedly was found to occur as early as the first cell division (127). This study highlights the power of using scRNA-seq and computational analyses to elucidate cell-fate decisions at the singe-cell level.

### Two Models of CD8 T Cell Differentiation

**Figure 4:**
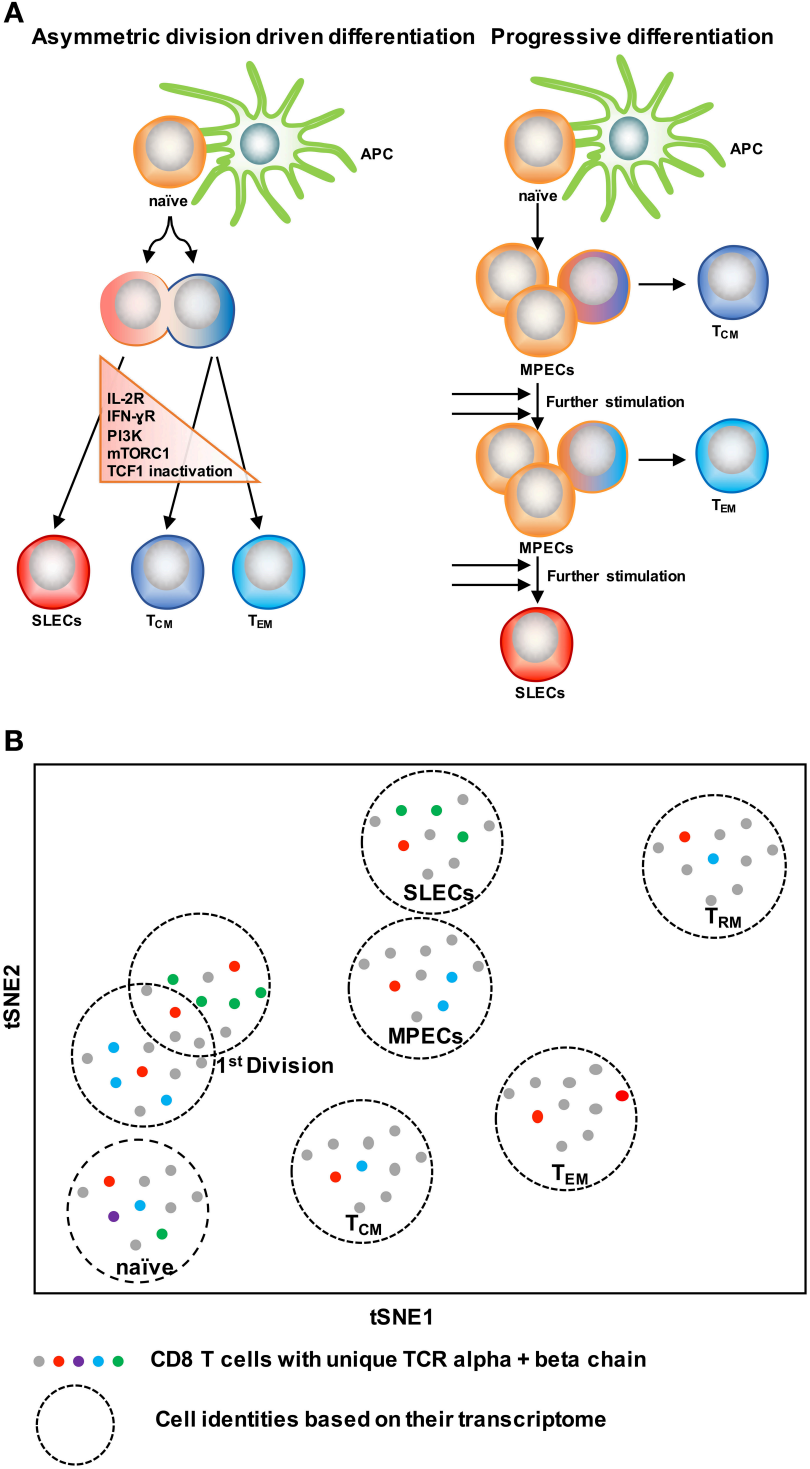
Two CD8 T cell differentiation models. **(A)** The asymmetric division model emphasizes the significance of asymmetric segregation of cytokine receptors and signals pathways as early as the first division in dictating the memory or effector potential of T cells. The proximal daughter cell (red) inherits molecules that make it more likely to become an effector cell, while the distal daughter (blue) inherits more memory-related molecules. The progressive differentiation model is a linear model in which the cumulative history of encounters with antigen and inflammation dictate the cell fate from a memory-like stage to terminally differentiated cells. **(B)** A method to depict “one cell, one fate” and “one cell, multiple fates” models. Single cell identity of a T cell can be profiled based on their transcriptome using scRNA-seq. TCRs are nature molecular tags to track T cells. Integration of TCR clonotypes to a gene expression profile on a single-cell level can monitor the dynamics of effect and memory CD8 T cell fate decision during infection.

With the knowledge of possible cellular fates (single fate vs. multiple fates) at the single cell level, the next question to ask is: how does this subset diversification occur following infection? There are two possibilities to support this: the model of asymmetric division driven differentiation vs. progressive differentiation model (140, 142) (Figure 4A). According to the asymmetric division model, the generation of long and short-lived progenies from a single precursor T cell occurs at the immediate onset of response, i.e., as early as the first cell division (143). Asymmetric segregation of cytokine receptors like IL-2Rα and IFN-γR and intracellular signaling pathways like PI3K and mTORC1 during mitosis (92, 139, 143–146) can cause proximal and distal daughter cells to have differential cytokine signaling that may lead them toward an effector or memory cell differentiation process, respectively. Supporting this, three groups (127, 138, 139) have found that at the single cell level, cellular bifurcation is possible during early rounds of cellular division in response to acute infection. On the other hand, the progressive differentiation model supports the subset diversification process from a single cell via a gradual differentiation process, from a memory-like stage to terminally differentiated cells, which is affected by the signaling strength of the signals that are received in the priming phase (115, 142). This model has been supported by a study (147) using unbiased mathematical model and probabilistic framework. It has been shown that a linear developmental pathway is responsible for cell fate diversification, that progresses from slowly proliferating memory precursors to the rapidly expanding effector population. However, none of these models alone can explain why during differentiation some cells take multiple fates while some show extreme bias toward a singular fate. On this note, it is important to consider that cellular differentiation is a dynamic process and can be accompanied by encountering stochastic initial priming events, which can make a difference in the fate of every single cell, depending on their reception and interpretation of various signals. In this respect, both T cell intrinsic factors like: signaling strength, co-stimulation, amount of cell intrinsic signaling molecules, the epigenetic landscape, and cellular metabolism and also cell extrinsic factors like: anatomical location to interact with APC, and inflammation can affect the fate of a single T cell undergoing differentiation (1, 148–150). Intriguingly, a recent finding that showed, depending on the developmental origin of naïve CD8 T cells: either fetal derived CD8 T cells or adult bone marrow derived CD8 T cells, can give rise to either memory-like CD8 T cells in adulthood or in the generation of naïve-like CD8 T cells, respectively (151). The diverse *in vivo* response generated at the single cell level during acute infections may potentially be a result of the recruitment of heterogeneous naïve CD8 T cells, a topic, which demands further research. In terms of technological advancement, it is now possible to do *in vivo* fate mapping of single naïve CD8 T cells in a way which was limited previously with the usage of cell number, while simultaneously accounting for the influence of TCR and more importantly to recapitulate an *in vivo* natural infection scenario without the reliance on adoptive transfer strategies.

ScRNA-seq has been emerged as an innovative platform to understand the cellular development and differentiation process (127, 152, 153). With the power of computational analysis, it also offers an assessment of the subset diversification and developmental trajectory in an unbiased manner without reliance on the preexisting knowledge of cellular types (153, 154). To understand single T cell fate and its kinship with subsequent progenies, it is ideal to trace the cell fate decision by using a natural T cell lineage barcode, the TCR sequence (155) (Figure 4B). Using TCR sequencing to uncover the identity of single T cells was limited with the determination of both TCR alpha and beta chain information in a single cell (156–158). With the use of more powerful algorithms, it is now possible to reconstruct TCR alpha-beta gene information from single cell RNA sequencing data and to couple the cellular identity of a T cell with its transcriptomic profile at the single cell level (159, 160). This approach can overcome the usage of TCR transgenic T cells and can allow for *in vivo* single cell fate mapping by observing and tracing thousands of single T cells simultaneously in a natural infection setting (152–154).

## Concluding Remarks

Current studies on genome-wide transcriptional and epigenetic changes during infection have revealed that DNA methylation, histone modifications and transcriptional signatures define CD8 T cell subsets and regulate CD8 T differentiation. Eventually, an identification of a core set of transcription factors or epigenetic regulatory molecules that can regulate memory formation could potentially be sufficient to help reprogram terminally differentiated CD8 T cells. Such findings will undoubtedly have an impact on T cell-based therapies and vaccine designs. Although the epigenetic patterns associated with distinct T cell subsets are starting to be unraveled, additional functional analyses are needed to further reveal the role of epigenetic modifying proteins and their relationship to key transcription factors that coordinately work together to determine cell-fate decisions. Moreover, as naïve CD8 T cells go through tremendous changes in their cell cycle, metabolism, cell signaling, and genetic landscape, it is starting to become well-appreciated that individual effector cells may acquire distinct cell fates, that as a whole results in the generation of a heterogeneous pool of memory T cells. While our current understanding of CD8 memory formation is derived from investigations using pooled cell populations to study cell fate decisions, recent technological advances in scRNA-seq and computational approaches hold great promise for deciphering the true transcriptional heterogeneity of individual CD8 T cells.

## Author Contributions

YC, RZ, DS, and AK wrote the manuscript. YC, RZ, and WC edited the manuscript.

### Conflict of Interest Statement

The authors declare that the research was conducted in the absence of any commercial or financial relationships that could be construed as a potential conflict of interest.

## References

[1] Kaech SM Cui W. Transcriptional control of effector and memory CD8+ T cell differentiation. Nat Rev Immunol. (2012) 12:749–61. 10.1038/nri3307 23080391 PMC4137483

[2] Chang JT Wherry EJ Goldrath AW. Molecular regulation of effector and memory T cell differentiation. Nat Immunol. (2014) 15:1104–15. 10.1038/ni.3031 25396352 PMC4386685

[3] Ahmed R Gray D. Immunological memory and protective immunity: understanding their relation. Science (1996) 272:54–60. 10.1126/science.272.5258.54 8600537

[4] Schluns KS Kieper WC Jameson SC Lefrancois L. Interleukin-7 mediates the homeostasis of naive and memory CD8 T cells *in vivo*. Nat Immunol. (2000) 1:426–32. 10.1038/80868 11062503

[5] Kaech SM Tan JT Wherry EJ Konieczny BT Surh CD Ahmed R. Selective expression of the interleukin 7 receptor identifies effector CD8 T cells that give rise to long-lived memory cells. Nat Immunol. (2003) 4:1191–8. 10.1038/ni1009 14625547

[6] Joshi NS Cui W Chandele A Lee HK Urso DR Hagman J. Inflammation directs memory precursor and short-lived effector CD8(+) T cell fates via the graded expression of T-bet transcription factor. Immunity (2007) 27:281–95. 10.1016/j.immuni.2007.07.010 17723218 PMC2034442

[7] Kaech SM Wherry EJ. Heterogeneity and cell-fate decisions in effector and memory CD8+ T cell differentiation during viral infection. Immunity (2007) 27:393–405. 10.1016/j.immuni.2007.08.007 17892848 PMC3431921

[8] Sarkar S Kalia V Haining WN Konieczny BT Subramaniam S Ahmed R. Functional and genomic profiling of effector CD8 T cell subsets with distinct memory fates. J Exp Med. (2008) 205:625–40. 10.1084/jem.20071641 18316415 PMC2275385

[9] Ibegbu CC Xu YX Harris W Maggio D Miller JD Kourtis AP. Expression of killer cell lectin-like receptor G1 on antigen-specific human CD8+ T lymphocytes during active, latent, and resolved infection and its relation with CD57. J Immunol. (2005) 174:6088–94. 10.4049/jimmunol.174.10.6088 15879103

[10] van Leeuwen EM de Bree GJ Remmerswaal EB Yong SL Tesselaar K ten Berge IJ. IL-7 receptor alpha chain expression distinguishes functional subsets of virus-specific human CD8+ T cells. Blood (2005) 106:2091–8. 10.1182/blood-2005-02-0449 15947093

[11] Bengsch B Spangenberg HC Kersting N Neumann-Haefelin C Panther E von Weizsacker F. Analysis of CD127 and KLRG1 expression on hepatitis C virus-specific CD8+ T cells reveals the existence of different memory T-cell subsets in the peripheral blood and liver. J Virol. (2007) 81:945–53. 10.1128/JVI.01354-06 17079288 PMC1797464

[12] Mackay LK Rahimpour A Ma JZ Collins N Stock AT Hafon ML. The developmental pathway for CD103(+)CD8+ tissue-resident memory T cells of skin. Nat Immunol. (2013) 14:1294–301. 10.1038/ni.2744 24162776

[13] Sheridan BS Pham QM Lee YT Cauley LS Puddington L Lefrancois L. Oral infection drives a distinct population of intestinal resident memory CD8(+) T cells with enhanced protective function. Immunity (2014) 40:747–57. 10.1016/j.immuni.2014.03.007 24792910 PMC4045016

[14] Masopust D Ha SJ Vezys V Ahmed R. Stimulation history dictates memory CD8 T cell phenotype: implications for prime-boost vaccination. J Immunol. (2006) 177:831–9. 10.4049/jimmunol.177.2.831 16818737

[15] Croom HA Denton AE Valkenburg SA Swan NG Olson MR Turner SJ. Memory precursor phenotype of CD8+ T cells reflects early antigenic experience rather than memory numbers in a model of localized acute influenza infection. Eur J Immunol. (2011) 41:682–93. 10.1002/eji.201040625 21264852

[16] Nolz JC Harty JT. Protective capacity of memory CD8+ T cells is dictated by antigen exposure history and nature of the infection. Immunity (2011) 34:781–93. 10.1016/j.immuni.2011.03.020 21549619 PMC3103642

[17] Obar JJ Jellison ER Sheridan BS Blair DA Pham QM Zickovich JM. Pathogen-induced inflammatory environment controls effector and memory CD8+ T cell differentiation. J Immunol. (2011) 187:4967–78. 10.4049/jimmunol.1102335 21987662 PMC3208080

[18] Herndler-Brandstetter D Ishigame H Shinnakasu R Plajer V Stecher C Zhao J. KLRG1(+) Effector CD8(+) T cells lose KLRG1, differentiate into all memory T cell lineages, and convey enhanced protective immunity. Immunity (2018) 48:716–29.e8. 10.1016/j.immuni.2018.03.015 29625895 PMC6465538

[19] Hamann D Baars PA Rep MH Hooibrink B Kerkhof-Garde SR Klein MR. Phenotypic and functional separation of memory and effector human CD8+ T cells. J Exp Med. (1997) 186:1407–18. 10.1084/jem.186.9.1407 9348298 PMC2199103

[20] Sallusto F Lenig D Forster R Lipp M Lanzavecchia A. Two subsets of memory T lymphocytes with distinct homing potentials and effector functions. Nature (1999) 401:708–12. 10.1038/44385 10537110

[21] Masopust D Vezys V Marzo AL Lefrancois L. Preferential localization of effector memory cells in nonlymphoid tissue. Science (2001) 291:2413–7. 10.1126/science.1058867 11264538

[22] Gebhardt T Wakim LM Eidsmo L Reading PC Heath WR Carbone FR. Memory T cells in nonlymphoid tissue that provide enhanced local immunity during infection with herpes simplex virus. Nat Immunol. (2009) 10:524–30. 10.1038/ni.1718 19305395

[23] Masopust D Choo D Vezys V Wherry EJ Duraiswamy J Akondy R. Dynamic T cell migration program provides resident memory within intestinal epithelium. J Exp Med. (2010) 207:553–64. 10.1084/jem.20090858 20156972 PMC2839151

[24] von Andrian UH Mackay CR. T-cell function and migration. Two sides of the same coin. N Engl J Med. (2000) 343:1020–34. 10.1056/NEJM200010053431407 11018170

[25] Reinhardt RL Khoruts A Merica R Zell T Jenkins MK. Visualizing the generation of memory CD4 T cells in the whole body. Nature (2001) 410:101–5. 10.1038/35065111 11242050

[26] Tussey L Speller S Gallimore A Vessey R. Functionally distinct CD8+ memory T cell subsets in persistent EBV infection are differentiated by migratory receptor expression. Eur J Immunol. (2000) 30:1823–9. 10.1002/1521-4141(200007)30:7<1823::AID-IMMU1823>3.0.CO;2-6 10940871

[27] Jameson SC Masopust D. Diversity in T cell memory: an embarrassment of riches. Immunity (2009) 31:859–71. 10.1016/j.immuni.2009.11.007 20064446 PMC2957815

[28] Mueller SN Gebhardt T Carbone FR Heath WR. Memory T cell subsets, migration patterns, and tissue residence. Annu Rev Immunol. (2013) 31:137–61. 10.1146/annurev-immunol-032712-095954 23215646

[29] Bouneaud C Garcia Z Kourilsky P Pannetier C. Lineage relationships, homeostasis, and recall capacities of central- and effector-memory CD8 T cells *in vivo*. J Exp Med. (2005) 201:579–90. 10.1084/jem.20040876 15710650 PMC2213051

[30] Sheridan BS Lefrancois L. Regional and mucosal memory T cells. Nat Immunol. (2011) 12:485–91. 10.1038/ni.2029 21739671 PMC3224372

[31] Schenkel JM Masopust D. Tissue-resident memory T cells. Immunity (2014) 41:886–97. 10.1016/j.immuni.2014.12.007 25526304 PMC4276131

[32] Masopust D Vezys V Wherry EJ Barber DL Ahmed R. Cutting edge: gut microenvironment promotes differentiation of a unique memory CD8 T cell population. J Immunol. (2006) 176:2079–83. 10.4049/jimmunol.176.4.2079 16455963

[33] Wakim LM Woodward-Davis A Bevan MJ. Memory T cells persisting within the brain after local infection show functional adaptations to their tissue of residence. Proc Natl Acad Sci USA. (2010) 107:17872–9. 10.1073/pnas.1010201107 20923878 PMC2964240

[34] Casey KA Fraser KA Schenkel JM Moran A Abt MC Beura LK. Antigen-independent differentiation and maintenance of effector-like resident memory T cells in tissues. J Immunol. (2012) 188:4866–75. 10.4049/jimmunol.1200402 22504644 PMC3345065

[35] Skon CN Lee JY Anderson KG Masopust D Hogquist KA Jameson SC. Transcriptional downregulation of S1pr1 is required for the establishment of resident memory CD8+ T cells. Nat Immunol. (2013) 14:1285–93. 10.1038/ni.2745 24162775 PMC3844557

[36] Steinbach K Vincenti I Kreutzfeldt M Page N Muschaweckh A Wagner I. Brain-resident memory T cells represent an autonomous cytotoxic barrier to viral infection. J Exp Med. (2016) 213:1571–87. 10.1084/jem.20151916 27377586 PMC4986533

[37] Kumar BV Ma W Miron M Granot T Guyer RS Carpenter DJ. Human tissue-resident memory T cells are defined by core transcriptional and functional signatures in lymphoid and mucosal sites. Cell Rep. (2017) 20:2921–34. 10.1016/j.celrep.2017.08.078 28930685 PMC5646692

[38] Ely KH Cookenham T Roberts AD Woodland DL. Memory T cell populations in the lung airways are maintained by continual recruitment. J Immunol. (2006) 176:537–43. 10.4049/jimmunol.176.1.537 16365448

[39] Gattinoni L Lugli E Ji Y Pos Z Paulos CM Quigley MF. A human memory T cell subset with stem cell-like properties. Nat Med. (2011) 17:1290–7. 10.1038/nm.2446 21926977 PMC3192229

[40] Gattinoni L Speiser DE Lichterfeld M Bonini C. T memory stem cells in health and disease. Nat Med. (2017) 23:18–27. 10.1038/nm.4241 28060797 PMC6354775

[41] Cieri N Oliveira G Greco R Forcato M Taccioli C Cianciotti B. Generation of human memory stem T cells after haploidentical T-replete hematopoietic stem cell transplantation. Blood (2015) 125:2865–74. 10.1182/blood-2014-11-608539 25736310

[42] Oliveira G Ruggiero E Stanghellini MT Cieri N D'Agostino M Fronza R. Tracking genetically engineered lymphocytes long-term reveals the dynamics of T cell immunological memory. Sci Transl Med. (2015) 7:317ra198. 10.1126/scitranslmed.aac8265 26659572

[43] Bottcher JP Beyer M Meissner F Abdullah Z Sander J Hochst B. Functional classification of memory CD8(+) T cells by CX3CR1 expression. Nat Commun. (2015) 6:8306. 10.1038/ncomms9306 26404698 PMC4667439

[44] Gerlach C Moseman EA Loughhead SM Alvarez D Zwijnenburg AJ Waanders L. The chemokine receptor CX3CR1 defines three antigen-experienced CD8 T cell subsets with distinct roles in immune surveillance and homeostasis. Immunity (2016) 45:1270–84. 10.1016/j.immuni.2016.10.018 27939671 PMC5177508

[45] Zinkernagel RM Doherty PC Restriction of *in vitro* T cell-mediated cytotoxicity in lymphocytic choriomeningitis within a syngeneic or semiallogeneic system. Nature (1974) 248:701–2. 10.1038/248701a0 4133807

[46] Doherty PC Zinkernagel RM. H-2 compatibility is required for T-cell-mediated lysis of target cells infected with lymphocytic choriomeningitis virus. J Exp Med. (1975) 141:502–7. 10.1084/jem.141.2.502 123002 PMC2190521

[47] Obar JJ Lefrancois L. Memory CD8+ T cell differentiation. Ann N Y Acad Sci. (2010) 1183:251–66. 10.1111/j.1749-6632.2009.05126.x 20146720 PMC2836783

[48] Daniels MA Teixeiro E. TCR signaling in T cell memory. Front Immunol. (2015) 6:617. 10.3389/fimmu.2015.00617 26697013 PMC4674549

[49] Chen L Flies DB. Molecular mechanisms of T cell co-stimulation and co-inhibition. Nat Rev Immunol. (2013) 13:227–42. 10.1038/nri3405 23470321 PMC3786574

[50] Hendriks J Xiao Y Rossen JW van der Sluijs KF Sugamura K Ishii N. During viral infection of the respiratory tract, CD27, 4-1BB, and OX40 collectively determine formation of CD8+ memory T cells and their capacity for secondary expansion. J Immunol. (2005) 175:1665–76. 10.4049/jimmunol.175.3.1665 16034107

[51] Boesteanu AC Katsikis PD. Memory T cells need CD28 costimulation to remember. Semin Immunol. (2009) 21:69–77. 10.1016/j.smim.2009.02.005 19268606 PMC2923542

[52] Bally AP Tang Y Lee JT Barwick BG Martinez R Evavold BD. Conserved region C functions to regulate PD-1 expression and subsequent CD8 T cell memory. J Immunol. (2017) 198:205–17. 10.4049/jimmunol.1601464 27895178 PMC5173413

[53] Sabins NC Chornoguz O Leander K Kaplan F Carter R Kinder M. TIM-3 engagement promotes effector memory T cell differentiation of human antigen-specific CD8 T cells by activating mTORC1. J Immunol. (2017) 199:4091–102. 10.4049/jimmunol.1701030 29127145 PMC5713500

[54] Badovinac VP Porter BB Harty JT. CD8+ T cell contraction is controlled by early inflammation. Nat Immunol. (2004) 5:809–17. 10.1038/ni1098 15247915

[55] Kolumam GA Thomas S Thompson LJ Sprent J Murali-Krishna K. Type I interferons act directly on CD8 T cells to allow clonal expansion and memory formation in response to viral infection. J Exp Med. (2005) 202:637–50. 10.1084/jem.20050821 16129706 PMC2212878

[56] Pearce EL Shen H. Generation of CD8 T cell memory is regulated by IL-12. J Immunol. (2007) 179:2074–81. 10.4049/jimmunol.179.4.2074 17675465

[57] Shaulov A Murali-Krishna K. CD8 T cell expansion and memory differentiation are facilitated by simultaneous and sustained exposure to antigenic and inflammatory milieu. J Immunol. (2008) 180:1131–8. 10.4049/jimmunol.180.2.1131 18178853

[58] Cui W Joshi NS Jiang A Kaech SM. Effects of Signal 3 during CD8 T cell priming: bystander production of IL-12 enhances effector T cell expansion but promotes terminal differentiation. Vaccine (2009) 27:2177–87. 10.1016/j.vaccine.2009.01.088 19201385 PMC2803112

[59] Wiesel M Crouse J Bedenikovic G Sutherland A Joller N Oxenius A. Type-I IFN drives the differentiation of short-lived effector CD8+ T cells *in vivo*. Eur J Immunol. (2012) 42:320–9. 10.1002/eji.201142091 22102057

[60] Weninger W Crowley MA Manjunath N von Andrian UH. Migratory properties of naive, effector, and memory CD8(+) T cells. J Exp Med. (2001) 194:953–66. 10.1084/jem.194.7.953 11581317 PMC2193483

[61] Berard M Brandt K Bulfone-Paus S Tough DF. IL-15 promotes the survival of naive and memory phenotype CD8+ T cells. J Immunol. (2003) 170:5018–26. 10.4049/jimmunol.170.10.5018 12734346

[62] Richer MJ Pewe LL Hancox LS Hartwig SM Varga SM Harty JT. Inflammatory IL-15 is required for optimal memory T cell responses. J Clin Invest. (2015) 125:3477–90. 10.1172/JCI81261 26241055 PMC4588296

[63] Kalia V Sarkar S Subramaniam S Haining WN Smith KA Ahmed R. Prolonged interleukin-2Ralpha expression on virus-specific CD8+ T cells favors terminal-effector differentiation *in vivo*. Immunity (2010) 32:91–103. 10.1016/j.immuni.2009.11.010 20096608

[64] Pipkin ME Sacks JA Cruz-Guilloty F Lichtenheld MG Bevan MJ Rao A. Interleukin-2 and inflammation induce distinct transcriptional programs that promote the differentiation of effector cytolytic T cells. Immunity (2010) 32:79–90. 10.1016/j.immuni.2009.11.012 20096607 PMC2906224

[65] Blattman JN Grayson JM Wherry EJ Kaech SM Smith KA Ahmed R. Therapeutic use of IL-2 to enhance antiviral T-cell responses *in vivo*. Nat Med. (2003) 9:540–7. 10.1038/nm866 12692546

[66] Cui W Liu Y Weinstein JS Craft J Kaech SM. An interleukin-21-interleukin-10-STAT3 pathway is critical for functional maturation of memory CD8+ T cells. Immunity (2011) 35:792–805. 10.1016/j.immuni.2011.09.017 22118527 PMC3431922

[67] El-Asady R Yuan R Liu K Wang D Gress RE Lucas PJ. TGF-{beta}-dependent CD103 expression by CD8(+) T cells promotes selective destruction of the host intestinal epithelium during graft-versus-host disease. J Exp Med. (2005) 201:1647–57. 10.1084/jem.20041044 15897278 PMC2212926

[68] Zhang N Bevan MJ. Transforming growth factor-beta signaling controls the formation and maintenance of gut-resident memory T cells by regulating migration and retention. Immunity (2013) 39:687–96. 10.1016/j.immuni.2013.08.019 24076049 PMC3805703

[69] Evans CM Jenner RG. Transcription factor interplay in T helper cell differentiation. Brief Funct Genomics (2013) 12:499–511. 10.1093/bfgp/elt025 23878131 PMC3838196

[70] Youngblood B Hale JS Kissick HT Ahn E Xu X Wieland A. (2017). Effector CD8 T cells dedifferentiate into long-lived memory cells. Nature 552:404. 10.1038/nature25144 29236683 PMC5965677

[71] Scott-Browne JP Lopez-Moyado IF Trifari S Wong V Chavez L Rao A. Dynamic changes in chromatin accessibility occur in CD8(+) T cells responding to viral infection. Immunity (2016) 45:1327–40. 10.1016/j.immuni.2016.10.028 27939672 PMC5214519

[72] Yu B Zhang K Milner JJ Toma C Chen R Scott-Browne JP Epigenetic landscapes reveal transcription factors that regulate CD8+ T cell differentiation. Nat Immunol. (2017) 18:573–82. 10.1038/ni.3706 28288100 PMC5395420

[73] Wang D Diao H Getzler AJ Rogal W Frederick MA Milner J. The transcription factor Runx3 establishes chromatin accessibility of cis-regulatory landscapes that drive memory cytotoxic T lymphocyte formation. Immunity (2018) 48:659–74.e6. 10.1016/j.immuni.2018.03.028 29669249 PMC6750808

[74] Russ BE Olshanksy M Smallwood HS Li J Denton AE Prier JE. Distinct epigenetic signatures delineate transcriptional programs during virus-specific CD8(+) T cell differentiation. Immunity (2014) 41:853–65. 10.1016/j.immuni.2014.11.001 25517617 PMC4479393

[75] Yao S Buzo BF Pham D Jiang L Taparowsky EJ Kaplan MH. Interferon regulatory factor 4 sustains CD8(+) T cell expansion and effector differentiation. Immunity (2013) 39:833–45. 10.1016/j.immuni.2013.10.007 24211184 PMC3855863

[76] Kurachi M Barnitz RA Yosef N Odorizzi PM DiIorio MA Lemieux ME. The transcription factor BATF operates as an essential differentiation checkpoint in early effector CD8+ T cells. Nat Immunol. (2014) 15:373–83. 10.1038/ni.2834 24584090 PMC4000237

[77] Godec J Cowley GS Barnitz RA Alkan O Root DE Sharpe AH Inducible RNAi *in vivo* reveals that the transcription factor BATF is required to initiate but not maintain CD8+ T-cell effector differentiation. Proc Natl Acad Sci USA. (2015) 112:512–7. 10.1073/pnas.1413291112 25548173 PMC4299213

[78] Xin G Schauder DM Lainez B Weinstein JS Dai Z Chen Y. A critical role of IL-21-induced BATF in sustaining CD8-T-cell-mediated chronic viral control. Cell Rep. (2015) 13:1118–24. 10.1016/j.celrep.2015.09.069 26527008 PMC4859432

[79] Nayar R Schutten E Bautista B Daniels K Prince AL Enos M Graded levels of IRF4 regulate CD8+ T cell differentiation and expansion, but not attrition, in response to acute virus infection. J Immunol. (2014) 192:5881–93. 10.4049/jimmunol.1303187 24835398 PMC4080788

[80] Man K Gabriel SS Liao Y Gloury R Preston S Henstridge DC. Transcription factor IRF4 promotes CD8(+) T cell exhaustion and limits the development of memory-like T cells during chronic infection. Immunity (2017) 47:1129–41.e5. 10.1016/j.immuni.2017.11.021 29246443

[81] Nowyhed HN Huynh TR Thomas GD Blatchley A Hedrick CC. Cutting edge: the orphan nuclear receptor Nr4a1 regulates CD8+ T cell expansion and effector function through direct repression of Irf4. J Immunol. (2015) 195:3515–9. 10.4049/jimmunol.1403027 26363057 PMC4592102

[82] Roychoudhuri R Clever D Li P Wakabayashi Y Quinn KM Klebanoff CA. BACH2 regulates CD8(+) T cell differentiation by controlling access of AP-1 factors to enhancers. Nat Immunol. (2016) 17:851–60. 10.1038/ni.3441 27158840 PMC4918801

[83] Kim MT Harty JT. Impact of inflammatory cytokines on effector and memory CD8+ T cells. Front Immunol. (2014) 5:295. 10.3389/fimmu.2014.00295 24995011 PMC4062963

[84] Danilo M Chennupati V Silva JG Siegert S Held W. Suppression of Tcf1 by inflammatory cytokines facilitates effector CD8 T cell differentiation. Cell Rep. (2018) 22:2107–17. 10.1016/j.celrep.2018.01.072 29466737

[85] Siegel AM Heimall J Freeman AF Hsu AP Brittain E Brenchley JM. A critical role for STAT3 transcription factor signaling in the development and maintenance of human T cell memory. Immunity (2011) 35:806–18. 10.1016/j.immuni.2011.09.016 22118528 PMC3228524

[86] Abdelsamed HA Moustaki A Fan Y Dogra P Ghoneim HE Zebley CC. Human memory CD8 T cell effector potential is epigenetically preserved during *in vivo* homeostasis. J Exp Med. (2017) 214:1593–606. 10.1084/jem.20161760 28490440 PMC5461005

[87] Dominguez CX Amezquita RA Guan T Marshall HD Joshi NS Kleinstein SH. The transcription factors ZEB2 and T-bet cooperate to program cytotoxic T cell terminal differentiation in response to LCMV viral infection. J Exp Med. (2015) 212:2041–56. 10.1084/jem.20150186 26503446 PMC4647261

[88] Guan T Dominguez CX Amezquita RA Laidlaw BJ Cheng J Henao-Mejia J. ZEB1, ZEB2, and the miR-200 family form a counterregulatory network to regulate CD8(+) T cell fates. J Exp Med. (2018) 215:1153–68. 10.1084/jem.20171352 29449309 PMC5881466

[89] Kim EH Sullivan JA Plisch EH Tejera MM Jatzek A Choi KY. Signal integration by Akt regulates CD8 T cell effector and memory differentiation. J Immunol. (2012) 188:4305–14. 10.4049/jimmunol.1103568 22467649 PMC3331885

[90] Rao RR Li Q Gubbels Bupp MR Shrikant PA. Transcription factor Foxo1 represses T-bet-mediated effector functions and promotes memory CD8(+) T cell differentiation. Immunity (2012) 36:374–87. 10.1016/j.immuni.2012.01.015 22425248 PMC3314246

[91] Kim MV Ouyang W Liao W Zhang MQ Li MO. The transcription factor Foxo1 controls central-memory CD8+ T cell responses to infection. Immunity (2013) 39:286–97. 10.1016/j.immuni.2013.07.013 23932570 PMC3809840

[92] Lin WW Nish SA Yen B Chen YH Adams WC Kratchmarov R. CD8(+) T lymphocyte self-renewal during effector cell determination. Cell Rep. (2016) 17:1773–82. 10.1016/j.celrep.2016.10.032 27829149 PMC5108530

[93] Gray SM Amezquita RA Guan T Kleinstein SH Kaech SM. Polycomb repressive complex 2-mediated chromatin repression guides effector CD8(+) T cell terminal differentiation and loss of multipotency. Immunity (2017) 46:596–608. 10.1016/j.immuni.2017.03.012 28410989 PMC5457165

[94] Jeannet G Boudousquie C Gardiol N Kang J Huelsken J Held W. Essential role of the Wnt pathway effector Tcf-1 for the establishment of functional CD8 T cell memory. Proc Natl Acad Sci USA. (2010) 107:9777–82. 10.1073/pnas.0914127107 20457902 PMC2906901

[95] Zhou X Yu S Zhao DM Harty JT Badovinac VP Xue HH. Differentiation and persistence of memory CD8(+) T cells depend on T cell factor 1. Immunity (2010) 33:229–40. 10.1016/j.immuni.2010.08.002 20727791 PMC2928475

[96] Utzschneider DT Delpoux A Wieland D Huang X Lai CY Hofmann M. Active maintenance of T cell memory in acute and chronic viral infection depends on continuous expression of FOXO1. Cell Rep. (2018) 22:3454–67. 10.1016/j.celrep.2018.03.020 29590615 PMC5942184

[97] Xing S Li F Zeng Z Zhao Y Yu S Shan Q. Tcf1 and Lef1 transcription factors establish CD8(+) T cell identity through intrinsic HDAC activity. Nat Immunol. (2016) 17:695–703. 10.1038/ni.3456 27111144 PMC4873337

[98] Hombrink P Helbig C Backer RA Piet B Oja AE Stark R. Programs for the persistence, vigilance and control of human CD8+ lung-resident memory T cells. Nat Immunol. (2016) 17:1467–78. 10.1038/ni.3589 27776108

[99] Mackay LK Minnich M Kragten NA Liao Y Nota B Seillet C. Hobit and Blimp1 instruct a universal transcriptional program of tissue residency in lymphocytes. Science (2016) 352:459–63. 10.1126/science.aad2035 27102484

[100] Mackay LK Kallies A. Transcriptional Regulation of Tissue-Resident Lymphocytes. Trends Immunol. (2017) 38:94–103. 10.1016/j.it.2016.11.004 27939451

[101] Milner JJ Goldrath AW. Transcriptional programming of tissue-resident memory CD8(+) T cells. Curr Opin Immunol. (2018) 51:162–9. 10.1016/j.coi.2018.03.017 29621697 PMC5943164

[102] Milner JJ Toma C Yu B Zhang K Omilusik K Phan AT. Runx3 programs CD8(+) T cell residency in non-lymphoid tissues and tumours. Nature (2017) 552:253–7. 10.1038/nature24993 29211713 PMC5747964

[103] Boddupalli CS Nair S Gray SM Nowyhed HN Verma R Gibson JA. ABC transporters and NR4A1 identify a quiescent subset of tissue-resident memory T cells. J Clin Invest. (2016) 126:3905–16. 10.1172/JCI85329 27617863 PMC5096804

[104] Zaid A Mackay LK Rahimpour A Braun A Veldhoen M Carbone FR. Persistence of skin-resident memory T cells within an epidermal niche. Proc Natl Acad Sci USA (2014) 111:5307–12. 10.1073/pnas.1322292111 24706879 PMC3986170

[105] Mackay LK Wynne-Jones E Freestone D Pellicci DG Mielke LA Newman DM. T-box transcription factors combine with the cytokines TGF-beta and IL-15 to control tissue-resident memory T cell fate. Immunity (2015) 43:1101–11. 10.1016/j.immuni.2015.11.008 26682984

[106] Ladle BH Li K-P Phillips MJ Pucsek AB Haile A Powell JD. *De novo* DNA methylation by DNA methyltransferase 3a controls early effector CD8^+^ T-cell fate decisions following activation. Proc Natl Acad Sci USA. (2016) 113:10631–6. 10.1073/pnas.1524490113 27582468 PMC5035851

[107] Carty SA Gohil M Banks LB Cotton RM Johnson ME Stelekati E. The loss of TET2 promotes CD8(+) T cell memory differentiation. J Immunol. (2018) 200:82–91. 10.4049/jimmunol.1700559 29150566 PMC5736442

[108] ENCODE Project Consortium Birney E Stamatoyannopoulos JA Dutta A Guigo R Gingeras TR. Identification and analysis of functional elements in 1% of the human genome by the ENCODE pilot project. Nature (2007) 447:799–816. 10.1038/nature05874 17571346 PMC2212820

[109] Heintzman ND Stuart RK Hon G Fu Y Ching CW Hawkins RD. Distinct and predictive chromatin signatures of transcriptional promoters and enhancers in the human genome. Nat Genet. (2007) 39:311–8. 10.1038/ng1966 17277777

[110] Heintzman ND Hon GC Hawkins RD Kheradpour P Stark A Harp LF. Histone modifications at human enhancers reflect global cell-type-specific gene expression. Nature (2009) 459:108–12. 10.1038/nature07829 19295514 PMC2910248

[111] Creyghton MP Cheng AW Welstead GG Kooistra T Carey BW Steine EJ. Histone H3K27ac separates active from poised enhancers and predicts developmental state. Proc Natl Acad Sci USA. (2010) 107:21931–6. 10.1073/pnas.1016071107 21106759 PMC3003124

[112] Buenrostro JD Giresi PG Zaba LC Chang HY Greenleaf WJ. Transposition of native chromatin for fast and sensitive epigenomic profiling of open chromatin, DNA-binding proteins and nucleosome position. Nat Methods (2013) 10:1213–8. 10.1038/nmeth.2688 24097267 PMC3959825

[113] He B Xing S Chen C Gao P Teng L Shan Q. CD8+ T Cells Utilize Highly Dynamic Enhancer Repertoires and Regulatory Circuitry in Response to Infections. Immunity (2016) 45:1341–54. 10.1016/j.immuni.2016.11.009 27986453 PMC5304416

[114] Pace L Goudot C Zueva E Gueguen P Burgdorf N Waterfall JJ. The epigenetic control of stemness in CD8+ T cell fate commitment. Science (2018) 359:177–86. 10.1126/science.aah6499 29326266

[115] Akondy RS Fitch M Edupuganti S Yang S Kissick HT Li KW. Origin and differentiation of human memory CD8 T cells after vaccination. Nature (2017) 552:362–7. 10.1038/nature24633 29236685 PMC6037316

[116] Fitzpatrick DR Shirley KM Kelso A Cutting edge: stable epigenetic inheritance of regional IFN-γ promoter demethylation in CD44^high^ CD8^+^ T lymphocytes. J Immunol. (1999) 162:5053–7. 10227972

[117] Lee PP Fitzpatrick DR Beard C Jessup HK Lehar S Makar KW. A critical role for Dnmt1 and DNA methylation in T cell development, function, and survival. Immunity (2001) 15:763–74. 10.1016/S1074-7613(01)00227-8 11728338

[118] Kersh EN Fitzpatrick DR Murali-Krishna K Shires J Speck SH Boss JM Rapid demethylation of the *IFN*-γ gene occurs in memory but not naive CD8 T cells. J Immunol. (2006) 176:4083–93. 10.4049/jimmunol.176.7.4083 16547244

[119] Northrop JK Thomas RM Wells AD Shen H. Epigenetic remodeling of the *IL-2* and *IFN*-γ loci in memory CD8 T cells is influenced by CD4 T cells. J Immunol. (2006) 177:1062–9. 10.4049/jimmunol.177.2.1062 16818762

[120] Scharer CD Barwick BG Youngblood BA Ahmed R Boss JM. Global DNA methylation remodeling accompanies CD8 T cell effector function. J Immunol. (2013) 191:3419–29. 10.4049/jimmunol.1301395 23956425 PMC3800465

[121] Northrop JK Wells AD Shen H. Cutting edge: chromatin remodeling as a molecular basis for the enhanced functionality of memory CD8 T cells. J Immunol. (2008) 181:865–8. 10.4049/jimmunol.181.2.865 18606637

[122] Fann M Godlove JM Catalfamo M Wood WH Chrest FJ Chun N. Histone acetylation is associated with differential gene expression in the rapid and robust memory CD8^+^ T-cell response. Blood (2006) 108:3363–70. 10.1182/blood-2006-02-005520 16868257 PMC1895425

[123] Araki Y Fann M Wersto R Weng NP. Histone acetylation facilitates rapid and robust memory CD8 T cell response through differential expression of effector molecules (Eomesodermin and its targets: Perforin and Granzyme B). J Immunol. (2008) 180:8102–8. 10.4049/jimmunol.180.12.8102 18523274 PMC2493419

[124] Denton AE Russ BE Doherty PC Rao S Turner SJ. Differentiation-dependent functional and epigenetic landscapes for cytokine genes in virus-specific CD8^+^ T cells. Proc Natl Acad Sci USA. (2011) 108:15306–11. 10.1073/pnas.1112520108 21876173 PMC3174616

[125] Araki Y Wang Z Zang C Wood WH III Schones D Cui K. Genome-wide analysis of histone methylation reveals chromatin state-based regulation of gene transcription and function of memory CD8+ T cells. Immunity (2009) 30:912–25. 10.1016/j.immuni.2009.05.006 19523850 PMC2709841

[126] Crompton JG Narayanan M Cuddapah S Roychoudhuri R Ji Y Yang W. Lineage relationship of CD8+ T cell subsets is revealed by progressive changes in the epigenetic landscape. Cell Mol Immunol. (2016) 13:502. 10.1038/cmi.2015.32 25914936 PMC4947817

[127] Kakaradov B Arsenio J Widjaja CE He Z Aigner S Metz PJ. Early transcriptional and epigenetic regulation of CD8(+) T cell differentiation revealed by single-cell RNA sequencing. Nat Immunol. (2017) 18:422–32. 10.1038/ni.3688 28218746 PMC5360497

[128] Rodriguez RM Suarez-Alvarez B Lavín JL Mosén-Ansorena D Baragaño Raneros A Márquez-Kisinousky L. Epigenetic networks regulate the transcriptional program in memory and terminally differentiated CD8^+^ T cells. J Immunol. (2017) 198:937–49. 10.4049/jimmunol.1601102 27974453

[129] Shin HM Kapoor V Guan T Kaech SM Welsh RM Berg LJ. Epigenetic modifications induced by Blimp-1 Regulate CD8(+) T cell memory progression during acute virus infection. Immunity (2013) 39:661–75. 10.1016/j.immuni.2013.08.032 24120360 PMC3808842

[130] Ciofani M Madar A Galan C Sellars M Mace K Pauli F. A validated regulatory network for Th17 cell specification. Cell (2012) 151:289–303. 10.1016/j.cell.2012.09.016 23021777 PMC3503487

[131] Johnson JL Georgakilas G Petrovic J Kurachi M Cai S Harly C. Lineage-determining transcription factor TCF-1 initiates the epigenetic identity of T cells. Immunity (2018) 48:243–57.e10. 10.1016/j.immuni.2018.01.012 29466756 PMC5824646

[132] Zhou X Xue HH. Cutting edge: generation of memory precursors and functional memory CD8+ T cells depends on T cell factor-1 and lymphoid enhancer-binding factor-1. J Immunol. (2012) 189:2722. 10.4049/jimmunol.1201150 22875805 PMC3437003

[133] Moon JJ Chu HH Pepper M McSorley SJ Jameson SC Kedl RM. Naive CD4(+) T cell frequency varies for different epitopes and predicts repertoire diversity and response magnitude. Immunity (2007) 27:203–13. 10.1016/j.immuni.2007.07.007 17707129 PMC2200089

[134] Jenkins MK Chu HH McLachlan JB Moon JJ. On the composition of the preimmune repertoire of T cells specific for Peptide-major histocompatibility complex ligands. Annu Rev Immunol. (2010) 28:275–94. 10.1146/annurev-immunol-030409-101253 20307209

[135] Stemberger C Huster KM Koffler M Anderl F Schiemann M Wagner H. A single naive CD8+ T cell precursor can develop into diverse effector and memory subsets. Immunity (2007) 27:985–97. 10.1016/j.immuni.2007.10.012 18082432

[136] Gerlach C van Heijst JW Swart E Sie D Armstrong N Kerkhoven RM. One naive T cell, multiple fates in CD8+ T cell differentiation. J Exp Med. (2010) 207:1235–46. 10.1084/jem.20091175 20479114 PMC2882844

[137] Buchholz VR Flossdorf M Hensel I Kretschmer L Weissbrich B Graf P. Disparate individual fates compose robust CD8+ T cell immunity. Science (2013) 340:630–5. 10.1126/science.1235454 23493420

[138] Plumlee CR Sheridan BS Cicek BB Lefrancois L. Environmental cues dictate the fate of individual CD8+ T cells responding to infection. Immunity (2013) 39:347–56. 10.1016/j.immuni.2013.07.014 23932571 PMC3817618

[139] Arsenio J Kakaradov B Metz PJ Kim SH Yeo GW Chang JT. Early specification of CD8+ T lymphocyte fates during adaptive immunity revealed by single-cell gene-expression analyses. Nat Immunol. (2014) 15:365–72. 10.1038/ni.2842 24584088 PMC3968536

[140] Reiner SL Sallusto F Lanzavecchia A. Division of labor with a workforce of one: challenges in specifying effector and memory T cell fate. Science (2007) 317:622–5. 10.1126/science.1143775 17673652

[141] Tubo NJ Pagan AJ Taylor JJ Nelson RW Linehan JL Ertelt JM. Single naive CD4+ T cells from a diverse repertoire produce different effector cell types during infection. Cell (2013) 153:785–96. 10.1016/j.cell.2013.04.007 23663778 PMC3766899

[142] Sallusto F Geginat J Lanzavecchia A. Central memory and effector memory T cell subsets: function, generation, and maintenance. Annu Rev Immunol (2004) 22:745–63. 10.1146/annurev.immunol.22.012703.104702 15032595

[143] Chang JT Palanivel VR Kinjyo I Schambach F Intlekofer AM Banerjee A. Asymmetric T lymphocyte division in the initiation of adaptive immune responses. Science (2007) 315:1687–91. 10.1126/science.1139393 17332376

[144] Chang JT Ciocca ML Kinjyo I Palanivel VR McClurkin CE Dejong CS. Asymmetric proteasome segregation as a mechanism for unequal partitioning of the transcription factor T-bet during T lymphocyte division. Immunity (2011) 34:492–504. 10.1016/j.immuni.2011.03.017 21497118 PMC3088519

[145] Lin WH Adams WC Nish SA Chen YH Yen B Rothman NJ. Asymmetric PI3K signaling driving developmental and regenerative cell fate bifurcation. Cell Rep. (2015) 13:2203–18. 10.1016/j.celrep.2015.10.072 26628372 PMC4685001

[146] Pollizzi KN Sun IH Patel CH Lo YC Oh MH Waickman AT. Asymmetric inheritance of mTORC1 kinase activity during division dictates CD8(+) T cell differentiation. Nat Immunol. (2016) 17:704–11. 10.1038/ni.3438 27064374 PMC4873361

[147] Buchholz VR Graf P Busch DH. The smallest unit: effector and memory CD8(+) T cell differentiation on the single cell level. Front Immunol. (2013) 4:31. 10.3389/fimmu.2013.00031 23424063 PMC3573211

[148] Rohr JC Gerlach C Kok L Schumacher TN. Single cell behavior in T cell differentiation. Trends Immunol. (2014) 35:170–7. 10.1016/j.it.2014.02.006 24657362

[149] Buchholz VR Schumacher TN Busch DH. T cell fate at the single-cell level. Annu Rev Immunol. (2016) 34:65–92. 10.1146/annurev-immunol-032414-112014 26666651

[150] Henning AN Roychoudhuri R Restifo NP. Epigenetic control of CD8(+) T cell differentiation. Nat Rev Immunol. (2018) 18:340–56. 10.1038/nri.2017.146 29379213 PMC6327307

[151] Smith NL Patel RK Reynaldi A Grenier JK Wang J Watson NB. Developmental origin governs CD8(+) T cell fate decisions during infection. Cell (2018) 174:117–130.e14. 10.1016/j.cell.2018.05.029 29909981

[152] Lonnberg T Svensson V James KR Fernandez-Ruiz D Sebina I Montandon R. Single-cell RNA-seq and computational analysis using temporal mixture modelling resolves Th1/Tfh fate bifurcation in malaria. Sci Immunol. (2017) 2:eaal2192. 10.1126/sciimmunol.aal2192 28345074 PMC5365145

[153] Zemmour D Zilionis R Kiner E Klein AM Mathis D Benoist C Single-cell gene expression reveals a landscape of regulatory T cell phenotypes shaped by the TCR. Nat Immunol. (2018) 19:291–301. 10.1038/s41590-018-0051-0 29434354 PMC6069633

[154] Guo X Zhang Y Zheng L Zheng C Song J Zhang Q Global characterization of T cells in non-small-cell lung cancer by single-cell sequencing. Nat Med. (2018) 24:978–85. 10.1038/s41591-018-0045-3 29942094

[155] Han A Glanville J Hansmann L Davis MM. Linking T-cell receptor sequence to functional phenotype at the single-cell level. Nat Biotechnol. (2014) 32:684–92. 10.1038/nbt.2938 24952902 PMC4337815

[156] Venturi V Quigley MF Greenaway HY Ng PC Ende ZS McIntosh T. A mechanism for TCR sharing between T cell subsets and individuals revealed by pyrosequencing. J Immunol. (2011) 186:4285–94. 10.4049/jimmunol.1003898 21383244

[157] Ji X Lyu SC Spindler M Bacchetta R Goncharov I Han A Deep profiling of single T cell receptor repertoire and phenotype with targeted RNA-seq (TECH2P.927). J Immunol. (2015) 194(1 Suppl.):206.237–206.237.

[158] Li B Li T Pignon JC Wang B Wang J Shukla SA. Landscape of tumor-infiltrating T cell repertoire of human cancers. Nat Genet. (2016) 48:725–32. 10.1038/ng.3581 27240091 PMC5298896

[159] Stubbington MJT Lonnberg T Proserpio V Clare S Speak AO Dougan G. T cell fate and clonality inference from single-cell transcriptomes. Nat Methods (2016) 13:329–32. 10.1038/nmeth.3800 26950746 PMC4835021

[160] Afik S Yates KB Bi K Darko S Godec J Gerdemann U. Targeted reconstruction of T cell receptor sequence from single cell RNA-seq links CDR3 length to T cell differentiation state. Nucleic Acids Res. (2017) 45:e148. 10.1093/nar/gkx615 28934479 PMC5766189

